# ‘Balancing Challenges and Personal Resources’: A Qualitative Study of Women's Experiences of Arm Impairment After Axillary Surgery for Breast Cancer

**DOI:** 10.1111/jan.16517

**Published:** 2024-10-07

**Authors:** Matilda Appelgren, Yvonne Wengström, Jana de Boniface, Helena Sackey

**Affiliations:** ^1^ Department of Molecular Medicine and Surgery Karolinska Institutet Stockholm Sweden; ^2^ Department of Surgery Capio St Göran's Hospital Stockholm Sweden; ^3^ Department of Neurobiology, Care Sciences and Society, Division of Nursing Karolinska Institutet Stockholm Sweden; ^4^ Karolinska Comprehensive Cancer Center Karolinska University Hospital Stockholm Sweden; ^5^ Department of Breast, Endocrine Tumors and Sarcoma Karolinska University Hospital Stockholm Sweden

**Keywords:** adaptation, affirmation, arm impairment, axillary surgery, breast cancer, focus group discussion, nursing, qualitative content analysis

## Abstract

**Aim:**

To explore how women previously treated for breast cancer experience living with arm impairment after axillary surgery.

**Design:**

Descriptive qualitative study. The inductive starting point for the analysis was followed by a deductive approach as the categories were related to the components of the sense of coherence framework.

**Methods:**

Twenty‐eight relapse‐free Swedish‐speaking females participated in six focus group discussions conducted between September and December 2022. All participants had undergone sentinel lymph node biopsy with or without completion axillary dissection 4 years earlier. Data were analysed using qualitative content analysis.

**Results:**

Three categories and an overall theme were identified. The categories ‘Sense‐making’, ‘Daily life’ and ‘Driving force’ reflect actions to understand and prevent arm symptoms, adaptations made in daily life and the empowering resources adopted to meet challenges. The overall theme, ‘Balancing challenges and personal resources’, comprised a process that began at diagnosis and remained ongoing for some participants. Most participants considered their new life situations manageable. However, those with more pronounced arm impairment reported that they did not always receive adequate aid, and that their daily lives were negatively affected.

**Conclusion:**

Returning to everyday life after axillary surgery for breast cancer is associated with varying degrees of challenges. Individuals with persistent arm impairment find returning to normal life more challenging. Therefore, further improvements in person‐centred care are of utmost importance.

**Patient and Public Contribution:**

Members of the Swedish Breast Cancer Association were involved in the creation of the interview guide.

**Impact:**

This study emphasises the requirement for providing further individualised support to those living with more severe arm impairment after axillary surgery.

**Reporting Method:**

This study was reported in accordance with the Standards for Reporting Qualitative Research.

**Summary**

- Impact statement
  ○ This study provides insights into the long‐term consequences of axillary surgery for women treated for breast cancer. It highlights the need for more personalised support for those experiencing severe arm impairment after axillary surgery. The findings may contribute to improved person‐centred preoperative information and follow‐up assessments, ultimately enhancing the quality of life for affected women. The study's implications extend to clinical practice, patient information and potentially reducing healthcare cost through better‐targeted information, interventions and support.
- What does this paper contribute to the wider global clinical community?
  ○ Provides a deeper understanding of the effects of arm impairment following axillary surgery on daily life.
  ○ Insights into the needs, adjustments and coping strategies of individuals with arm impairment after axillary surgery.
  ○ Offers important knowledge that may further improve person‐centred care.

## 1 Introduction

The clinical manifestations of arm impairment, a known consequence of axillary lymph node dissection (ALND) in breast cancer, may include lymphedema, pain, numbness, tingling and limited arm and shoulder movements (Appelgren et al. 2022; Bartels et al. 2023; Sackey et al. 2014; Vrancken Peeters et al. 2023). Sentinel lymph node biopsy (SLNB) is a less extensive surgical method for axillary staging owing to the removal of fewer lymph nodes. Consequently, the frequency and severity of arm impairment following SLNB are significantly lower (Appelgren et al. 2022; Sackey et al. 2014; Vrancken Peeters et al. 2023). For patients with 1–2 metastases in the SLNB, axillary radiotherapy is a safe alternative to completion ALND (Bartels et al. 2023; de Boniface et al. 2024). Nevertheless, approximately 14% of all individuals with breast cancer in Sweden continue to undergo ALND (Confederation of Regional Cancer Centers in Sweden [RCC] 2023a) despite the widespread trend of adopting less extensive axillary surgery.

Breast cancer is the most common type of cancer affecting women globally, with approximately 2.3 million cases being diagnosed annually (Bray et al. 2024). As a result, many women are living with the consequences of axillary surgery.

## 2 Background

In research aiming to reduce the extent of axillary surgery, both patient‐reported outcome measures (PROMs) and objective measures are used to determine arm impairment and health‐related quality of life (HRQoL). The exact incidence of arm impairment after axillary surgery is difficult to determine; however, some degree of arm impairment has been observed in 13%–40% of patients undergoing ALND (Appelgren et al. 2022; Bartels et al. 2023; Galimberti et al. 2018; Naoum et al. 2020; Sackey et al. 2014), and in 8%–10% of patients undergoing SLNB alone (Appelgren et al. 2022; Naoum et al. 2020). Notably, the overall HRQoL did not seem to differ despite the severity of arm impairment being significantly lesser when ALND was omitted (Appelgren et al. 2022; Bartels et al. 2023). Nevertheless, arm impairment may affect daily life, manifesting as difficulty in returning to work, increased prevalence of mental health issues and reduced engagement in social and physical activities (Appelgren et al. 2022; Nesvold et al. 2010; Zomkowski et al. 2018).

For women treated for breast cancer, the experience of living with arm impairment after axillary surgery is underexplored. In addition to analysing objective measures and PROMs that describe and evaluate arm symptoms, detailed individual experiences of living with the consequences of axillary surgery can only be explored using qualitative methods. Qualitative research methods facilitate a deeper understanding of how individuals respond to these situations and capture the variations in multilayered experiences (Graneheim and Lundman 2004; Krueger and Casey 2015, 21). Predominantly, qualitative studies have described the experiences of breast cancer survivors following breast cancer treatment. However, to the best of our knowledge, only one qualitative study has explored the experiences of living with arm impairment after breast cancer surgery. This study, published in 2009, revealed that arm impairment has negative impact on work, household activities and family relations (Thomas‐Maclean et al. 2009). A subsequent analysis based on the same population explored arm impairment in relation to leisure activities and reported different adaptations, such as performing activities at a slower pace and taking breaks when needed (Thomas et al. 2015).

Follow‐up programmes for individuals with breast cancer aim to assess and manage the long‐term physical and psychosocial effects of breast cancer treatment, in addition to identifying local recurrence or contralateral breast cancer (RCC 2023b; Runowicz et al. 2016). Annual follow‐up with breast imaging for at least 5 years, combined with either clinical visits or contact by letter or phone, is recommended by the Swedish national guidelines (RCC 2023b). Remote contact limits the objective assessment of arm impairment; thus, the manner of framing the questions as well as receiving and interpreting the answers plays an important role in the evaluation of potential arm impairment and further planning of care and support.

The improvements in breast cancer survival rates over the last few decades (Siegel et al. 2023) indicate the importance of exploring how individuals experience living with persistent negative consequences of axillary surgery. A deeper understanding will facilitate the formulation of follow‐up care that enables proper assessment of arm impairment and adequate support. This study reports the results of six focus group discussions exploring women's experiences of living with arm‐related impairment after axillary surgery for breast cancer.

## 3 The Study

### 3.1 Aim

To explore how women previously treated for breast cancer experience living with arm impairment after axillary surgery.

### 3.2 Design

This study used a descriptive qualitative study design where data were collected from focus group discussions followed by a qualitative content analysis. The inductive starting point aims to describe the varying experiences and perceptions (Graneheim and Lundman 2004) without using a preconceived model or hypothesis (Elo and Kyngäs 2008). However, a deductive approach (Eriksson and Lindström 1997) based on the emerging patterns of the categories related to the components of the sense of coherence (SOC) framework (Antonovsky 1987) was used later in the analysis. The SOC framework was added to the analysis abductively to facilitate the description and understanding of the findings (Eriksson and Lindström 1997).

### 3.3 Study Setting and Recruitment

Swedish‐speaking women who had been operated for breast cancer within the randomised SENOMAC trial in 2018 (de Boniface et al. 2017) and had no recurrence were eligible for inclusion in this present study. The participants with sentinel lymph node‐positive breast cancer were randomised to undergo completion ALND or omit this procedure in the SENOMAC trial. In addition, the participants underwent annual mammography and clinical on‐site visits (de Boniface et al. 2017). Clinical on‐site visits were temporarily discontinued and replaced by remote contact during the Coronavirus disease 2019 (COVID‐19) pandemic.

Purposeful sampling (Patton 2015, 265) was performed to achieve a balanced distribution of age and geographic residential area among the participants. An even distribution of participants who had undergone completion ALND versus SLNB alone was sought, irrespective of the degree of reported arm impairment. Ninety‐seven eligible participants were sent an invitation letter and informed consent form by their study sites. Among the 33 participants who returned signed informed consent forms, 28 participated in the focus group discussions. One individual withdrew consent, and four individuals dropped out, including one owing to an online connection failure.

### 3.4 Data Collection

Data were collected throughout six focus group discussions between September and December 2022. All participants had undergone breast cancer surgery 4 years prior. Focus group discussions were conducted to benefit from the dynamics among the participants as they discussed their experiences (Krueger and Casey 2015, 41). All discussions were conducted on a password‐secured Zoom platform (Zoom Video Communications, Inc.). The participants were instructed to describe their experiences and expectations related to their axillary surgery by, first summarising of what they remembered from the early postoperative period (up to approximately 6 months postoperatively) and then focusing on their experiences today. The discussion was conducted according to a semistructured interview guide; probing questions were posed for clarification (Supporting Information 1). The number of participants in the focus group discussion ranged from 3 to 6 including one discussion leader and one moderator per group. Thus, three discussion groups with participants who had undergone SLNB only and three groups with participants who had undergone completion ALND were obtained. However, one participant with the opposite axillary staging method participated on two occasions; this was noted and left untreated. The duration of the group discussions ranged from 55 to 90 min. All sessions were audio‐recorded and transcribed verbatim. The participants also completed a short questionnaire on the nature of breast cancer surgery, additional treatment, level of education and social demographics.

### 3.5 Data Analysis

The data were analysed inspired by Graneheim and Lundman's (2004) model for qualitative content analysis. Table 1 presents examples of the analytical process. The analysis was conducted in a stepwise manner. The authors (MA, YW and HS) first read and re‐read the transcribed material to familiarise themselves with the data. The first author (MA) identified, condensed and sorted the meaning units based on their content. The manifest content of the meaning units was labelled with codes. With the research question in mind, the codes were repeatedly compared with the meaning units, and differences and similarities were discussed by the authors (MA, YW and HS). Subcategories and their categories were created via abstraction (Lindgren, Lundman, and Graneheim 2020). Once the categories were created, a deductive profile was adopted using the SOC framework (Antonovsky 1987) as an overarching description of the categories. The concordance between the subcategories and categories was reviewed based on the level of abstraction and guidance from the SOC framework domains and adjusted until a consensus was reached. Finally, the underlying meaning of the focus group discussions was interpreted as an overall theme.

**TABLE 1:** Illustration of the analysis process.

Illustration of the analysis process			
Condensed meaning unit	Code	Subcategory	Category
When I got my treatment, after the operation, I started doing exercise and I've continued with that … Exercise was good for me. (1:5)	Recovery	Transition	Daily life
But there was that thing with the numbness, I mean that it went numb … but that has decreased over time. (4:4)	Healing process		
Even if I can't stretch out my arm to reach things as nimbly as before … I've gotten used to that, so it's not something that bothers me in my everyday life. (5:2)	Reorientation		
Yes, I have exactly the same thing, it feels stiff in my armpit and on the back across my arm. (6:5)	Physical symptoms	Physical and psychological impact	
I always get help when I want to get things off the top shelves. And I'm not as strong, I don't carry as much, that's something I've noticed. (3:1)	Adjusting activities		
I find it a real hassle to deal with clothes when you have one pale, fat arm and one that's normal. (2:5)	Body image		
My arm problems have been really difficult for me at times, they've made me feel pretty lousy. (4:5)	Psychological suffering		

*Note:* Numbers in parenthesis indicate the focus group number followed by the numeric ID of the participant.

### 3.6 Ethical Considerations

The study was conducted according to the principles of the Declaration of Helsinki (World Medical Association 2013) and was approved by the Swedish Ethical Review Authority (Ref. 2022‐01416‐02, 2014/1165‐31/1). Written informed consent was obtained from all participants and they were informed that participation was voluntary and could be withdrawn at any time.

### 3.7 Rigour and Reflexivity

Similar to in‐person focus group discussions, discussions conducted on digital platforms enable the collection of rich personal descriptions of experiences and attitudes (Woodyatt, Finneran, and Stephenson 2016). The use of a digital platform in this study facilitated the participation of women from different parts of Sweden. No more than six participants were included in each discussion group to improve rigour and credibility in data collection, as recommended by Lander et al. (2023). The authors (MA and HS) found the data rich and mature for analysis by the sixth interview. The method of qualitative content analysis described by Graneheim and Lundman (2004) and Lindgren, Lundman, and Graneheim (2020) facilitated a structured stepwise analysis. Authentic quotes are presented as examples of the original data from which the codes were created. The first steps of the analysis were undertaken in Swedish; however, a switch to English was made once the codes and categories emerged. The quotes presented in section four and meaning unit examples presented in Table 1 were translated from Swedish into English by a certified translator.

The manifest content of the text, that is, the voices of the participants, was actively focused on during the analysis to address the potential influence of the authors' preunderstanding, based on their longstanding clinical experience in breast cancer treatment. A scrutinising approach was used with the aim to ensure that the assumptions emerging from the analysis stemmed solely from the participants' information. This was achieved by triangulating the findings of the analysis with the coauthors, and continuously reviewing the emerging codes, subcategories and categories until a consensus was reached. The authors actively reflected on whether their preunderstanding affected the analysis after each analysis section. The first author (MA) repeatedly returned to the transcribed data and scrutinised the credibility of the emerged codes and subcategories to ensure representativeness.

The focus group discussion leaders were only presented by name and as researchers during the data collection; no information regarding their clinical occupation was revealed. The discussion leader and the moderator had no clinical or therapeutic relationships with the participants. Each focus group was followed by a methodological discussion led by coauthor YW, who has extensive experience in focus group discussions, to further enhance the rigour of the data collection.

The codes containing data unrelated to the aim of the study were not classified into subcategories. This content was related to investigation, postoperative complications, support during chemotherapy and the isolated consequences of chemotherapy, such as hand and foot neuropathies. This study was reported in accordance with the Standards for Reporting Qualitative Research guidelines (O'Brien et al. 2014).

## 4 Findings

Six focus group discussions were conducted in this study. Twenty‐eight participants with 1–2 nodal metastases who had undergone SLNB only (*n* = 10) or SLNB plus completion ALND (*n* = 18) 4 years ago were recruited from 13 different geographic areas in Sweden. Sixteen participants had undergone breast‐conserving surgery, and the remaining 12 participants underwent mastectomy with or without reconstruction. The median age of the participants at the time of the interviews was 58 years (range 43–80). Two‐thirds of the participants shared a household and had a university level education. Table 2 presents the complete demographic and treatment characteristics.

**TABLE 2:** Demographic and treatment characteristics.

Variable	Categories	*N* = 28
Age^a^	Median age (min–max)	58 (43–80)
	< 50 years	8 (29)
	50–65 years	13 (46)
	> 65 years	7 (25)
Type of breast surgery		
	BCS	16 (57)
	Mastectomy	12 (43)
Type of axillary surgery		
	SLNB only	10 (36)
	ALND	18 (64)
Chemotherapy		
	Yes	26 (96)
	No	1 (4)
	Missing	1
Endocrine therapy^b^		
	Yes	22 (79)
	No	6 (21)
Radiotherapy		
	Yes	27 (96)
	No	1 (4)
Geographic area of residency		
	Large city (> 200.000 inhabitants)	9 (32)
	Intermediate city (> 50.000 inhabitants)	9 (32)
	Village (< 50.000 inhabitants)	10 (36)
Country of childhood		
	Sweden	24 (86)
	Other	4 (14)
Working status		
	Working	19 (68)
	Sick leave^c^	2 (7)
	Retired	7 (25)
Social status		
	Living alone	7 (25)
	Share household	21 (75)
Level of education		
	Primary school	1 (4)
	High school	6 (21)
	University	21 (75)

*Note:* Presented as numbers and percentages if not stated otherwise.

Abbreviations: ALND, axillary lymph node dissection; BCS, breast‐conserving surgery; SLNB, sentinel lymph node biopsy.

^a^ Age by the time of interview.

^b^ Ongoing breast cancer–related endocrine therapy.

^c^ Any percentage of sick leave.

The analysis yielded three categories, ‘Sense‐making’, ‘Daily life’ and ‘Driving force’, in addition to one overall theme, ‘Balancing challenges and personal resources’ (Table 3). The coping strategies and resources reported by the participants were related to the three interrelated components of the SOC framework: comprehensibility, manageability and meaningfulness. The SOC framework reflects the ability of a person to cope with stressors in everyday life. Comprehensibility is defined as the ability to understand, explain and predict life events. Manageability is defined as the availability of sufficient resources to meet expected or unexpected events. Meaningfulness, a motivational component, is defined as the ability of an individual to gain emotional understanding and motivation to engaging with difficulties, that is, feeling that it is worth the effort to face the different problems and demands encountered in life (Antonovsky 1987). For instance, the participants of the present study gained comprehensibility by learning about arm impairment, enhanced manageability through different self‐care actions and adaptations, and meaningfulness was created via positive thinking and affirmation.

**TABLE 3:** Overview of the findings.

Subcategory	Category	Framework	Theme
Understanding Preparedness	Sense‐making	Comprehensibility	‘Balancing challenges and personal resources’
Transition Physical and psychological impact	Daily life	Manageability	
Positive thinking Affirmation Perseverance	Driving force	Meaningfulness	

### 4.1 Balancing Challenges and Personal Resources

The overall theme, the balance between challenges and personal resources, began at diagnosis and continued through the healing and recovery phases, although less prominently, until today. The following sections elucidate the resources used by the participants to balance the challenges they faced, divided by the assigned category.

#### 4.1.1 Sense‐Making

The participants described that both they and their family members wished to understand how and why symptoms of arm impairment arouse, learn how to relieve discomfort and prevent arm impairment in the future. This knowledge was obtained from healthcare providers and via internet searches and conversations with friends and others receiving treatment for the same condition. Overall, the participants described that they received useful postoperative advice regarding recovery and how to avoid arm impairment in the future. The diagnosis of breast cancer, combined with the overwhelming amount of information and language barriers, could hinder the intake of preoperative and postoperative information. Later, the advice was sometimes perceived as insufficient by participants with persistent arm impairment. These participants sought additional information from brochures and leaflets, their medical records and their contact nurses, whose availability and aid were greatly appreciated. Perceived arm impairment after the axillary surgery was expected; however, the participants expressed that they were less prepared for late‐onset symptoms.

So, I had been told that you could get lymphedema. And then I got an exercise program, but that was more about not becoming stiff, maintaining your arm mobility, and lifting it above your head and all that. Um, behind your back and that. Um, no, I do not think I can say that I was prepared for the possibility that it might become like this. I was not–not this much, anyway. (6:1)

#### 4.1.2 Daily Life

The recovery process, including postoperative wound healing, pain‐relief medications and extent of arm impairment, was considered manageable or better than expected. The participants actively participated in their own recovery and returned to work part‐time or full‐time. Furthermore, they reintroduced physical activities such as walking, running, going to the gymnasium, golfing and cycling early into their recovery process. The exercises recommended by healthcare providers aided in regaining shoulder and arm mobility.

Arm impairment was experienced by most participants to some extent. Most symptoms subsided over time; however, some participants who had undergone completion ALND had persistent or worsening arm impairments. Symptoms such as pain, burning sensation, numbness and feeling of soft swollen tissue in the armpit could persist for longer than expected and were still present for some. A decrease in arm strength and endurance were impairments commonly experienced years postsurgery. Arm impairment was constant in some cases, whereas others described pain or insufficient arm endurance only when exercising or carrying objects.

I got this burning sensation in the skin on my arm. So that when I put on some garment or if someone happened to brush against me, it was just as if I had a burn injury that they were touching. And it took a pretty long time for that sensation to go away–it took more than a year. (2:1)

Adjustments in daily life owing to arm impairment ranged from no adjustment to significant adjustments. Current activities that required adaptation included sleeping position, working hours and physical exercise. Restricted arm and shoulder mobility was addressed with stretching exercises, placing objects on the lower shelves in cupboards and asking for aid. Symptoms from the armpit and tight chest wall scars were experienced as limiting factors for arm and shoulder mobility, and the affected participants had to accept the necessity of regular arm stretching to prevent shoulder joint stiffness. Participants with a swollen arm required clothing adjustments, with some switching their wedding rings and watches from the affected hand or wrist to the opposite side.

I have split all my blouses, and I can hardly wear [laughter] any jackets. I have to search and search to find something so that I can get my arm into the jacket sleeve … and I know that I have to go to at least 15 different stores before I can find a jacket, for example, that fits my arm. (4:5)

Various preventive precautions were taken owing to concerns regarding the development of arm impairment in the future. Some participants avoided lifting heavy objects and performing physically demanding work, such as drilling, digging in the garden or working in commercial kitchens. Others with a swollen arm described specific preventive measures such as wearing a compression sleeve at all times, visiting a physiotherapist regularly or performing arm‐specific exercises habitually. The level of concern and precautions decreased over time, and at the time of the study, most participants did not spend a substantial amount of time thinking about their arm impairment. However, those with persistent arm impairment were negatively affected and had to accept these permanent concerns.

But now I have accepted it and so now, the first thing I do when I get up is get that sock on and get that glove on… I have to wear it when I go in the sea, or the pool or… I have to wear it pretty much 24 hours a day. And I have gotten used to it now and I accept it. There are some days when I feel “I cannot take it.” (4:5)

#### 4.1.3 Driving Force

Adaptation to and acceptance of arm impairment required mental strength, motivation and perseverance. Participants faced challenges, such as accepting bodily changes, unchanged expectations from work and society, difficulty in finding help for arm impairment and experienced physical and mental setbacks. However, they described adopting positive thinking, such as beliefs in destiny and purpose, feelings of hope and trust that everything will resolve or acceptance of life whichever way it turns out.

So that somehow, I think, well, but you have to see the positive side and believe that there is meaning in everything. Otherwise, you just sort of go under. I guess that is my way of adapting to the whole situation, really. That there has to be some meaning to it. And then of course maybe you cannot quite find it [laughter], but you have to try anyway. (3:3)

The positive outcomes of physical activity, such as increased arm mobility, elevated mood, having fun and being social, motivated the participants. However, not all participants had the motivation or energy to be physically active.

Being seen as an individual and receiving affirmation increased the motivation and perseverance of the participants. For example, through reliable, trusting and flexible follow‐up contacts with healthcare providers, individual needs were identified and responded to, which made the participants feel seen and affirmed. In addition to standard care, participation in the SENOMAC trial was mentioned as a valuable, complementary affirming activity.

… and they have encouraged me to do physical activity. And I mean that is another way of telling me that ‘Well, but your life will continue, you know’ [laughter], a bit of psychology, psychology. (6:4)

Some participants expressed that they felt misunderstood or abandoned. A lack of scheduled healthcare contacts or missed follow‐up visits was also described, and some participants with arm impairment felt that their symptoms and needs were not always being listened to or understood. Consequently, some participants talked to others with breast cancer experience, or actively sought care elsewhere to cope; however, some were uncertain that it would be worth the effort.

Finally, the participants expressed that they felt fortunate compared to others with greater arm impairment. Their experience of being diagnosed with breast cancer made them mentally stronger and more prepared for future challenges. Moreover, they expressed pride in having coped with the different treatments, adapting to a new life situation and finding their own experiences with breast cancer treatment worth sharing.

## 5 Discussion

The present study demonstrates that returning to everyday life while experiencing arm impairment is a process associated with varying degrees of challenges. Participants made adaptations and accepted their new life situation; however, those experiencing more pronounced arm impairment sometimes encountered difficulties in receiving adequate assistance. These participants did not always feel listened to and some experienced negative impacts on their daily lives.

Coping with arm impairment, as depicted in the overall theme of the analysis, comprised continuously balancing the available resources with the different demands of everyday life. This is a transformative and multidimensional process that involves knowledge, self‐care and motivation.

The present study explored the experiences of living with arm impairment among women 4 years after undergoing surgery for breast cancer, showing that the multidimensional process of adapting to life with arm impairment commenced at diagnosis and surgery, is still ongoing to this day. Thus, it can be considered an extended postoperative phase that eventually transitions to a stable everyday life in most cases. This observation is supported by the somewhat similar findings reported by Nilsson et al. (2020), who explored late postoperative experiences among patients who underwent general, hand or orthopaedic surgery. These experiences included the requirement for healthcare information, use of self‐care strategies for symptom management, receiving help and support from family and friends and finding motivation and perseverance.

Understanding the side effects of axillary surgery was crucial and actively pursued by the participants. A similar finding was observed in the study by Rosenberg, Butow, and Shaw (2021) which elucidated the coping strategies used by breast cancer survivors to manage belated treatment‐related side effects.

Adopting strategies, such as implementing modified behaviours, being physically active, and practising self‐care were described as ways to manage the recovery process and the challenges that daily life entailed. These strategies have also been identified in previous studies involving women treated for breast cancer (Drageset, Lindstrøm, and Underlid 2016; Rosenberg, Butow, and Shaw 2021). Empowering resources such as positive thinking, hobbies and work have been described in the present study and in studies conducted by Drageset, Lindstrøm, and Underlid (2016) and Llewellyn, Howard, and McCabe (2019). Being acknowledged as individuals and receiving affirmation strengthened the motivation and perseverance of the participants, especially when they faced challenges or setbacks. Building a relationship based on trust with healthcare professionals is important, this finding is consistent with those of other studies (Llewellyn, Howard, and McCabe 2019; Tsianakas et al. 2012).

The patterns of coping strategies described, and the resources used in this study were related to the components of the SOC framework: comprehensibility, manageability and meaningfulness. The SOC framework has been used to explain how individuals with cancer manage their situation (Strang and Strang 2001). Moreover, it has been shown to predict the use of coping strategies following breast cancer diagnosis (Kenne Sarenmalm et al. 2013).

The extent of arm impairment varied among the participants; however, most participants described their situation as manageable. In addition to the use of active coping strategies by the participants, this finding may also be attributed to a phenomenon known as response shift. A response shift is a natural change in the internal values and standards of a person occurring over time, that is, the same treatment‐related symptoms are perceived as milder or more manageable than before (Sprangers and Schwartz 1999).

The present study did not aim to compare the two axillary staging methods, SLNB only and completion ALND. However, some experiences related to more severe arm impairment that were unique to those who underwent completion ALND were identified in this study. These findings are to be expected, considering the earlier 1‐year PROM data evaluation from the SENOMAC trial, where the participants who had undergone completion ALND reported significantly worse arm symptoms than those in the SLNB‐only group (Appelgren et al. 2022).

In summary, the consistency of the coping strategies and resources described in the present study with those reported by previous studies indicates that this is a transformative process and that the resources used to manage the side effects of axillary surgery are similar to those used throughout breast cancer recovery.

### 5.1 Strengths and Limitations

The present study has several strengths. The methodology used, focus group discussions and qualitative content analysis provided new insights, thereby facilitating a multifaceted understanding of the complex phenomenon of adaptation to arm impairment after axillary surgery. Furthermore, the use of an online setting facilitated the inclusion of participants from 13 residencies in Sweden, encompassing rural and urban areas. Consistent with the findings of Woodyatt, Finneran, and Stephenson (2016), the rigour in data collection is not lost with the use of an online setting. The participants spoke freely, interacted with each other via nods of agreement and laughter, echoed similar sentiments and actively listened to and expanded upon the contributions of the other participants. The outbreak of the COVID‐19 pandemic and subsequent introduction of online meeting tools made many participants comfortable with the online format, and only one connection failure occurred among the four participants who dropped out. Lastly, the number of participants per focus group recommended by Lander et al. (2023) was adhered to, which further strengthened the rigour and credibility of data collection. Thus, the trustworthiness of the present study was increased.

Our findings need to be considered in light of some limitations. The experiences of the participants regarding the impact of arm impairment on daily life may have been influenced by their overall experience with the trajectory of breast cancer recovery. However, the participants described and discussed specific strategies for coping with arm impairment, such as understanding arm impairment, and integrating arm‐specific adaptations into daily routines, and performing arm‐specific self‐care actions. Moreover, the aim of the present study was to achieve an equal number of participants who had undergone SLNB only and completion ALND; however, this goal was not achieved. This may be attributed to the individuals who had undergone ALND being more motivated to share their experiences owing to a greater incidence of arm impairment. Similarly, the low response rate may indicate that the participants of the present study belong to a selected group who were more motivated to share their stories. The high education levels of the participants and their lower median age indicate that the study population could be more prone to adopting adaptive coping strategies (Bottaro and Faraci 2022). Thus, the findings of the present study might indicate a more positive situation than reality.

Nevertheless, we consider that the findings of the present study are based on rich and diverse descriptions of the experiences shared by the participants, facilitating the exploration of various and multilayered aspects of adaptation to arm impairment.

### 5.2 Implications for Practice

A strong trend towards de‐escalating axillary surgery has been observed in recent decades; however, approximately 14% of all individuals with breast cancer in Sweden continue to undergo ALND (RCC, 2023a). Additionally, SLNB is not completely without risk for developing arm impairment (Appelgren et al. 2022; Bartels et al. 2023; Naoum et al. 2020). Arm impairment may persist over time; for instance, the participants described a need to continue performing regular arm and shoulder exercises to counteract movement restrictions. This observation is supported by the findings of a study wherein restricted arm mobility was identified as a daily problem for up to 10 years postsurgery (Hauerslev et al. 2020).

Some adjustments in daily life were related to changes in autonomy and bodily appearance. These changes correspond to the physical function and body image domains in the HRQoL questionnaires by the European Organisation for Research and Treatment of Cancer (Aaronson et al. 1993; Sprangers et al. 1996). This observation may indicate a potential impact on HRQoL, which aligns with the findings of the present study, wherein participants with long‐lasting and more pronounced arm impairment experienced a negative impact on daily life. Thus, those individuals should receive priority from healthcare providers for preventive measures and support.

Gaining knowledge and awareness regarding the various long‐term consequences of axillary surgery is of high importance for both healthcare professionals and affected women. Individuals with persistent arm impairment can be identified, supported and allocated appropriate resources by conducting targeted follow‐up care assessments. In addition, asking about arm impairment, exploring the need for knowledge and supporting personal resources, person‐centred care and individually adapted support can be further improved.

## 6 Conclusion

The findings of this study provide a deeper understanding of the consequences of axillary surgery experienced by women treated for breast cancer. These insights may contribute to improved person‐centred preoperative information and follow‐up assessments. Returning to everyday life was a process associated with varying degrees of challenges, as evidenced by the experiences described by the participants, wherein those with persistent arm impairment experienced more challenges and limitations in their daily lives. Therefore, it is valuable to identify these individuals and offer them additional support, which may be facilitated by implementing objective measures and asking arm‐specific follow‐up questions in addition to using predefined PROMs.

## Author Contributions

All authors made substantial contributions to conception and design, or acquisition of data or analysis and interpretation of data; involved in drafting the manuscript or revising it critically for important intellectual content; given final approval of the version to be published. Each author should have participated sufficiently in the work to take public responsibility for appropriate portions of the content; agreed to be accountable for all aspects of the work in ensuring that questions related to the accuracy or integrity of any part of the work are appropriately investigated and resolved.

## Ethics Statement

The study has been approved by the Swedish Ethical Review Authority (Ref. 2022‐01416‐02, 2014/1165‐31/1).

## Consent

Written informed consent was obtained from all participants prior to data collection.

## Conflicts of Interest

The authors declare no conflicts of interest.

### Peer Review

The peer review history for this article is available at https://www.webofscience.com/api/gateway/wos/peer‐review/10.1111/jan.16517.

## Supporting information


**Data S1.** Interview guide.

## Data Availability

The data that support the findings of this study are available from the corresponding author upon reasonable request.
