# Supplementary material for: ‘Balancing Challenges and Personal Resources’: A Qualitative Study of Women's Experiences of Arm Impairment After Axillary Surgery for Breast Cancer
Source: J Adv Nurs. 2024 Oct 7;81(6):3156–65. doi: 10.1111/jan.16517 (PMC12080084; doi:10.1111/jan.16517)
Supplement: Supplementary file 1 — Data S1. Interview guide. [file JAN-81-3156-s001.docx]

Supplementary 1: Interview guide

| **Question areas** | **Interview Questions** |
| --- | --- |
| The post-operative experience (up to approximately six months after breast- and axillary surgery). | - Can you tell us something about your everyday experiences of the early post-operative period? - What was your self-care program? - How did you find your recovery? - Describe your everyday life in relation to your arm symptoms. - What were your abilities and your limitations after the armpit (axillary) surgery? |
| Your current experiences with symptoms/consequences of the axillary surgery. | - Describe your current everyday life in relation to your arm symptoms. - What are your symptoms from the arm and armpit? - Have you needed to do anything particular to deal with your arm symptoms, and if so, what? - Please describe if, and if so in what way, your arm symptoms had an impact on your everyday life, for example work, household tasks or leisure activities. - What were your expectations of the armpit (axillary) surgery? Did it turn out the way you thought? - Do you believe that you adapt to your symptoms over time? - If you are comfortable about sharing this: can you tell us if these problems have had an impact on your psychological wellbeing? |
| Since you are participants of the SENOMAC trial | - Do you want to share with us what made you participate in that study? |
